# Analysis of the genes and mechanisms responsible for the cytotoxicity of the *Legionella* Ofk308 strain in the *Tetrahymena* host

**DOI:** 10.3389/fmicb.2025.1643556

**Published:** 2025-08-18

**Authors:** Istiana Nur Vidayanti, Kenta Watanabe, Ema Fujino, Takashi Shimizu, Masahisa Watarai

**Affiliations:** ^1^Laboratory of Veterinary Public Health, The Joint Graduate School of Veterinary Medicine, Yamaguchi University, Yamaguchi, Japan; ^2^Laboratory of Veterinary Public Health, Joint Faculty of Veterinary Medicine, Yamaguchi University, Yamaguchi, Japan

**Keywords:** cytotoxic, host-pathogen interaction, *Legionella-*containing vacuole, *Legionella pneumophila*, *Tetrahymena*

## Abstract

**Background:**

*Legionella pneumophila*, an intracellular pathogen responsible for the pneumonia-like Legionnaires’ disease in humans, inhabits aquatic environments, including man-made water systems such as water fountains, foot spas, and tap water, and exists as part of biofilms or as a protozoan parasite. As a bacterivore, *Tetrahymena thermophila* provides a favorable environment for *Legionella* to establish a replicative niche (*Legionella*-containing vacuole; LCV) under environmental stress. Conversely, the *L. pneumophila* Ofk308 strain, isolated from an Ashiyu foot spa, has been found to be cytotoxic to the ciliate *T. thermophila* CU427. This study aimed to identify the cytotoxicity-related genes of *Legionella* and elucidate their mechanisms specific to the *Tetrahymena* host.

**Methods:**

A comparative analysis using RNA-sequencing was conducted with two *Legionella* strains, Philadelphia-1 and Ofk308, to select several candidate genes. Deletion mutants of Ofk308 were constructed by homologous recombination. Eight out of ten candidate gene deletion mutants were successfully generated. These mutants were analyzed for cytotoxicity against *T. thermophila* and intracellular bacterial growth at 2 h, 24 h, and 48 h postinfection.

**Results and Discussions:**

Among the deletion mutants, *Δ* vicinal oxygen chelate (VOC) and msrB/A exhibited reduced cytotoxicity. Furthermore, LCVs formed in *T. thermophila* infected with DVOC and msrB/A were smaller in size compared to those formed by the parental strain Ofk308, suggesting a role in both cytotoxicity and intracellular growth. Multiple factors contribute to the cytotoxicity exhibited by the Ofk308 strain in protozoan host cells, and gene expression analysis may reveal additional relevant factors.

## Introduction

*Legionella pneumophila*, a Gram-negative intracellular bacterium that infects and replicates within alveolar macrophages, causes Legionnaires’ disease or Pontiac fever. *L. pneumophila* Philadelphia-1 is firstly isolated after an explosive pneumonia outbreak in 1976 in Philadelphia. The Legionnaires’ mortality rate is ranging from 1 to 80% depends on risk factor such as age, transmission factor, and immunity. In the other hand, the Pontiac fever is considered as self-limiting flu-like disease*. Legionella* infects humans through aerosol inhalation, although human-to-human transmission is generally not established (Diederen, 2008; Yao et al., 2024). *L. pneumophila in*habits both natural and man-made aquatic ecosystems, such as water fountains, foot spas, and cooling towers (Head et al., 2021). Well-known as a pathogen with a broad range of protozoan hosts—*Paramecium* (Watanabe et al., 2016), *Acanthamoeba* (Kim et al., 2023), and *Tetrahymena thermophila*—the latter has emerged as a favorable host for *L. pneumophila*. Moreover, *L. pneumophila* is efficiently ingested but poorly digested by the ciliate *T. thermophila*. Coculturing this ciliate with *L. pneumophila* increases the survival and rapid multiplication of *Tetrahymena* (Berk et al., 2008; Boamah et al., 2017). These protozoan hosts are likely responsible for the environmental persistence of *Legionella* by providing a platform for survival and protection from various environmental stresses.

As previously reported, the *L. pneumophila* Ofk308 strain, an environmental isolate, exhibits cytotoxicity toward the *Paramecium* host (Watanabe et al., 2016). *Paramecium* infected with Ofk308 undergoes cell lysis and avoids phagosome–lysosome fusion (Watanabe et al., 2016). However, its cytotoxicity toward *T. thermophila* remains unclear. The findings of the study with *Tetrahymena* are important in clarifying whether the cytotoxicity of Ofk308 strain is very specific to *Paramecium* or whether it is widely exerted in other protozoan hosts. The Ofk308 strain harbors several well-characterized virulence factors, including *dot, lvh, rtx*, and *hsp60* (Tachibana et al., 2013). Notably, the Dot/Icm type IV secretion system is essential for intracellular replication, allowing *Legionella* to form a replication-permissive compartment, i.e., the *Legionella-*containing vacuole (LCV), via this system (Chauhan and Shames, 2021). We have also identified several other bacterial factors involved in interactions with protozoan hosts and analyzed their functions (Watanabe et al., 2016, 2018; Nishida et al., 2018). Interestingly, however, when comparing the gene profiles of general *Legionella* strains capable of utilizing protozoa as hosts with those of the cytotoxic Ofk308 strain, no marked differences were observed in the presence of these known genes. These findings suggest that an analysis focusing on expression levels or the timing of expression is required. In this study, we explored factors relatively highly expressed in the Ofk308 strain using RNA-seq and hypothesized that they may be novel candidate factors involved in cytotoxicity toward the protozoan host. Among the identified candidates, *fosA1,* encoding a putative vicinal oxygen chelate (VOC) protein family, and *msrA1,* encoding a bifunctional methionine sulfoxide reductase B/A (msrB/A), were found to contribute to cytotoxicity in *T. thermophila.* Understanding the interaction between these organisms from a public health perspective may help identify new environmental infection risks and control strategies.

## Materials and methods

### Bacterial strains and culture conditions

*Legionella* strain Ofk308 (accession number: BBUH0000000) comprises a genome with 38.2% G + C content and a total length of 3,473,188 bp and is closely related to the reference genome strain Philadelphia-1 (Phil-1) (Watanabe et al., 2015). Phil-1 (GTC_00296) and Ofk308 were maintained as frozen 20% glycerol stocks. The bacteria were cultured at 37°C on buffered charcoal yeast extract agar (BCYE) supplemented with 2.5% Fe and 4% L-cysteine or on a similar medium without agar and charcoal (AYE). *Escherichia coli* DH5α pir strain was cultured in Luria-Bertani (LB) liquid medium or on LB containing 1.5% agar. When required, antibiotics were added to the *Legionella* cultures at the following concentrations: kanamycin (30 μg/mL) and chloramphenicol (10 μg/mL).

### *T. thermophila* culture and maintenance

All *T. thermophila* strains, including CU427, were provided by the National BioResource Project, Yamaguchi University. They were maintained in proteose peptone (SPP) medium supplemented with FeCl_3_ at 30°C and passaged every 48–72 h.

### RNA-seq analysis

Phil-1 and Ofk308 strains were cultured at 37°C to the exponential phase in AYE, then diluted and cultured again at 30°C for 24 h. After culturing, total RNA was prepared using the Maxwell RSC Simply RNA Tissue Kit (Promega). Following rRNA removal with the NEBNext rRNA Depletion Kit (New England BioLabs), the NEBNext Ultra II Directional RNA Library Prep Kit for Illumina (New England BioLabs) was used with 20 ng of total RNA for library construction. Libraries were sequenced on an Illumina NovaSeq 6,000. The raw reads were mapped onto the Genomic Contig Format (GCF)_001753085.1 *L. pneumophila* sequence. Comparative expression analysis was performed using the RNA-Seq analysis tool of CLC Genomics Workbench.

### Deletion mutant bacteria strains construction

Eight deletion mutants were constructed through homologous recombination by cloning two polymerase chain reaction (PCR) fragments into Sal *I-*cleaved pSR47s, as previously described (Watanabe et al., 2018). PCR fragments were obtained by amplifying 1,500 bp upstream of the 5′ and downstream of the 3′ ends of the targeted gene in the parental strain Ofk308 using KOD-Plus-Neo Polymerase (the primer list is in Table 1). A plasmid carrying the upstream and downstream regions of the gene candidates was transformed into *E. coli* DH5α *λ* pir and then electroporated into Ofk308 using a Gene Pulser electroporator (Bio-Rad Laboratories, CA, United States) in 10% cold glycerol. A complementary strain was constructed using pNT562 (Nishida et al., 2017) carrying the VOC and msrB/A genes using the same method. Transformants were selected by culturing on BCYE agar plates containing kanamycin (30 μg/mL) and counter-selected on medium supplemented with 5% sucrose. Successful deletion mutant strains were confirmed by PCR using Tks Gflex DNA Polymerase.

**Table 1:** Primer lists.

Target region		Base
mscL_up	F	TATCGATACCGTCGATTCCAGGAATTGCTGTTTTTG
	R	TGATAGGGATGTCTGAATTGGCTGGCAAAAATAAAC
mscL_down	F	CAGACATCCCTATCATGATT
	R	ATCCTCTAGAGTCGATTTTCCAGCATTAGAAAACCG
lpofk_02643_up	F	TATCGATACCGTCGATTAAGTAACGCTTCGCTGAA
	R	TAGTGAGATGTGCTAATTTAAGTTCCTTTCTACAAGC
lpofk_02643_down	F	TAGCACATCTCACTAAAAGAC
	R	ATCCTCTAGAGTCGATCTGATATTAAAAATGAAAAATTT
msrA1_up	F	TATCGATACCGTCGACACAAAAAAACACACGCCC
	R	TCAAGGAGCATGAATTGTTCCGGAAGATTTAATCT
msrA1_down	F	TTCATGCTCCTTTGAAAACTC
	R	ATCCTCTAGAGTCGACCAATAGCGTCAATGGCAT
fosA_up	F	TATCGATACCGTCGACGATAGCGAGATACAAGCT
	R	GAACAAGGAATCATGAAATACGGCGGGGTTCCT
fosA_down	F	CATGATTCCTTGTTCATGTC
	R	ATCCTCTAGAGTCGAATAATTTTCTAGTTGTTCTCAC
lpofk_01237_up	F	TATCGATACCGTCGAATTATTAACAATACGCATACTTT
	R	AGAAAAATAGTCATTTGCAATGAAATTTCCGGCAA
lpofk_01237_down	F	AATGACTATTTTTCTATGAATAAA
	R	ATCCTCTAGAGTCGAGTCAAATATTTACAAACTTATAAA
lpofk_02264_up	F	TATCGATACCGTCGATAAGCTGCTCCATTCACCT
	R	ACAGATGCCTTTGAAACTCCTTAACAGGTATTAATG
lpofk_02264_down	F	TTCAAAGGCATCTGTGAGTG
	R	ATCCTCTAGAGTCGACTAACGTTCATAAGAGCTCC
lpofk_00673_up	F	TATCGATACCGTCGAAAAACTAATAGAGAAATTCATGC
	R	AGCCTTTTAAAGGAAAATATTCTCCATTTGTTGCAC
lpofk_00673_down	F	TTCCTTTAAAAGGCTTATTTGATCAAAATAAG
	R	ATCCTCTAGAGTCGAAACTCAAATCCCAGCTTGG
fosA_comp	F	CCCCCTCGAGGTCGAATGAGTATACAATTGAATCATAC
	R	TATCGATACCGTCGATTAAGACCCGCTACCATAAG
msrA1_comp	F	CCCCCCTCGAGGTCGAATGAGTGACTACCTCGATAA
	R	TATCGATACCGTCGACTAGCCAAAACGATCGACT
lpofk_02002_up	F	TATCGATACCGTCGAATCCAGGAGTGACTTGGC
	R	CAAGGAGTTAGTTACTTTACAGTAATGAAATGAGTTTT
lpofk_02002_down	F	GTAACTAACTCCTTGTTATCT
	R	ATCCTCTAGAGTCGAATTATTATTCTTTATGTTTTACTG
lpofk_00237_up	F	TATCGATACCGTCGATTTCAGTTGTTGGTTCGCC
	R	TCCCTCTCAATTTCCAATAATCTCTCCGCAAATGTG
lpofk_00237_down	F	GGAAATTGAGAGGGAAAAGC
	R	ATCCTCTAGAGTCGAATAAATACATTTATGACTTTATGG
ankX_2_up	F	TATCGATACCGTCGACAAATTGGGAGACAACGATT
	R	TGGCTGGAGGCCTGCAATAAATAGGCGGTATCAAATT
ankX_2_down	F	GCAGGCCTCCAGCCAGCG
	R	ATCCTCTAGAGTCGAATGTAAATCAAGTTATAGAAATAG
lpofk_01167_up	F	TATCGATACCGTCGACATCTGAAAATGGTTCATATTG
	R	TCTCACAAACTCCATGATGAATAACCTCATATTTTATT
lpofk_01167_down	F	ATGGAGTTTGTGAGATTTTGT
	R	ATCCTCTAGAGTCGATGTCCTCGATTAACTCATTAT

### Infection assays

Postexponential phase (48 h), *Legionella* cultures were used to infect *T. thermophila* (48 h after the last passage) at multiplicities of infection (MOIs) of 10,000, 1,000, and 100 and incubated at 30°C. The relative number of viable *Tetrahymena* cells was measured using a hemacytometer after fixation with 4% paraformaldehyde (PFA) at 2, 24, and 48 h postinfection (hpi).

### Intracellular growth assay

Forty-eight hours after the last passage, the *Tetrahymena* were infected with *Legionella* culture at an MOI of 10,000 and incubated at 30°C. At two h postinfection (hpi), extracellular bacteria were removed by gentle filtration-washing with SPP medium through a 10-μm pore-size membrane filter. The supernatant was resuspended in SPP medium to recover *Tetrahymena* that had fed on bacteria. Serial dilutions were performed to culture intracellular bacterial colony-forming units (CFU) on BCYE at 2, 24, and 48 hpi.

### Fluorescence microscopy

*Legionella* Phil-1, Ofk308, ΔVOC and msrB/A carrying pAM239-GFP was induced with isopropyl-*β*-D-thiogalactopyranoside (IPTG) (1 mM) in BYE medium and grown until the postexponential phase at 37°C. *Legionella* was then used to infect *Tetrahymena* at an MOI of 10,000. Subsequently, 4% paraformaldehyde in phosphate-buffered saline was used to fix the samples at 24–25°C for 5 min, and intracellular bacterial load was analyzed under a fluorescence microscope at 2, 24, and 48 hpi. LysoTracker (Invitrogen) was used to evaluate the maturation of *Tetrahymena* phagosomes containing either Phil-1 or Ofk308 strains at 30 and 120 min postinfection. Fluorescence images were captured using a Fluoview FV100 confocal laser scanning microscope at 100× magnification.

### Data analyses

All data were obtained from three identical experiments, and the error bars indicate the standard error. The results were statistically analyzed using GraphPad Prism software version 10. Two-way analysis of variance was employed to assess the effects of strain type (wild type and mutant) and MOI levels (10,000, 1,000, and 100) on the survival rate of CU427, intracellular growth, and LCV formation.

## Results

### Confirmation of cytotoxicity of Ofk308 to *Tetrahymena* host cells

**Figure 1:**
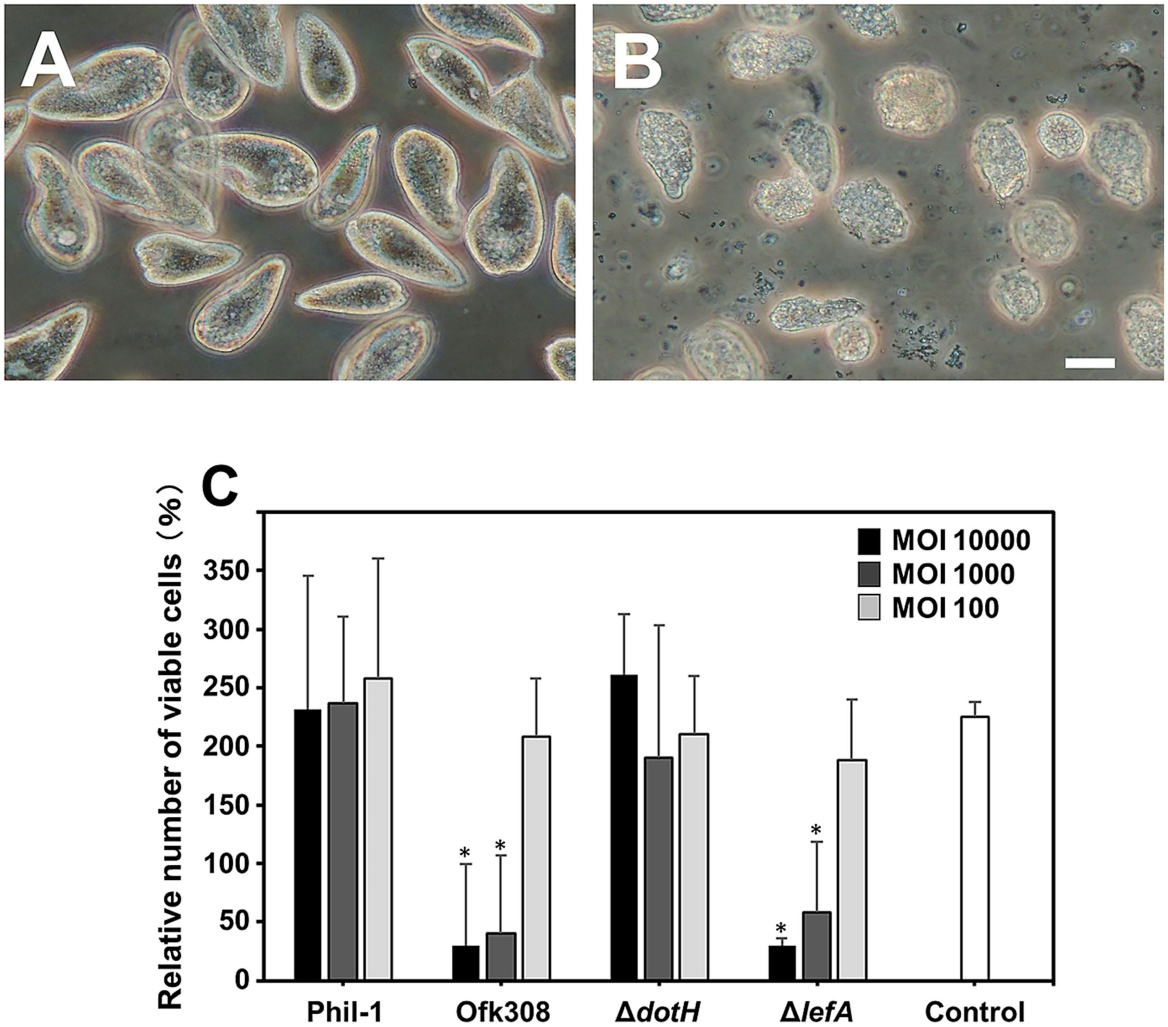
*Tetrahymena thermophila* CU427 cells were infected with *Legionella pneumophila* strain Philadelphia-1 (Phil-1) **(A)** or strain Ofk308 **(B)** at a multiplicity of infection (MOI) of 10,000, and cells were observed after 48 h. Scale bar, 20 μm. The number of viable CU427 cells within 48 h postinfection at various MOIs is shown in panel **(C)**. The number of viable CU427 cells are presented as relative values, with the number of cells at the start set to 100%. Statistically significant differences compared to Phil-1 are indicated by asterisks (*p* < 0.01).

We previously demonstrated that an environmentally isolated *L. pneumophila* strain, Ofk308, is cytotoxic to *Paramecium* spp., a protozoan host of *Legionella* (Watanabe et al., 2016). To assess whether similar cytotoxicity occurs in the *Tetrahymena* host, we conducted infection experiments. At 48 hpi, *Tetrahymena* cells infected with Ofk308 showed abnormal morphology and reduced viable cell numbers (Figures 1A,B). In contrast, the Phil-1 strain used as a control and the Ofk308 *dotH*-deletion mutant did not display these cytotoxic effects. The Ofk308 *lefA*-deletion mutant, which is less cytotoxic in *Paramecium* host cells (Watanabe et al., 2016), exhibited cytotoxicity similar to that of the parental Ofk308 strain (Figure 1C).

### Deletion mutant strains and *Tetrahymena thermophila* CU427 survival

**Figure 2:**
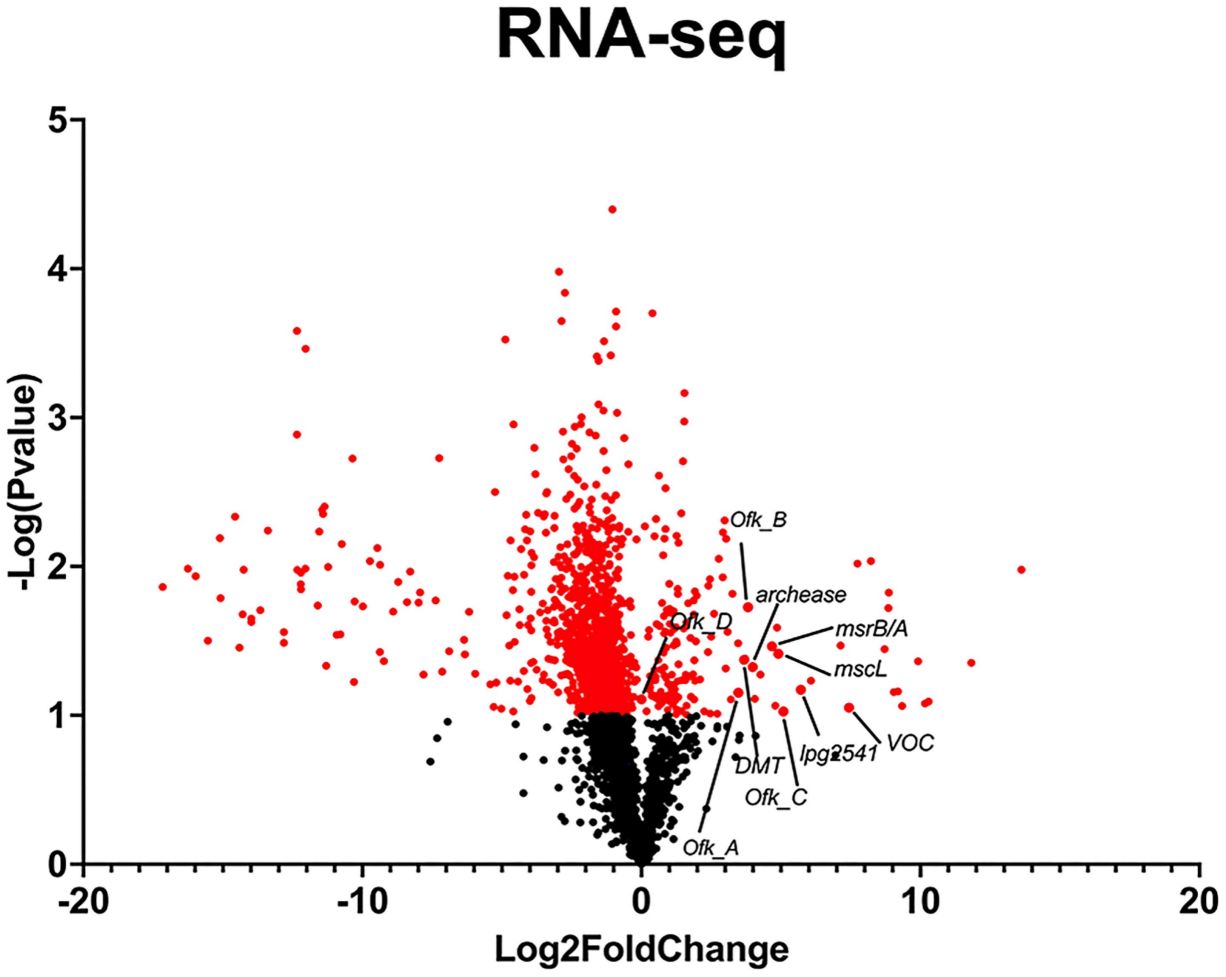
RNA-Seq analysis of *L. pneumophila* strains Ofk308 and Philadelphia-1 (Phil-1) for candidate gene selection. Fold change was calculated as the difference in gene expression between Ofk308 and Phil-1, normalized to Phil-1. A student’s *t*-test was used to determine statistical significance (*p* < 0.05).

**Figure 3:**
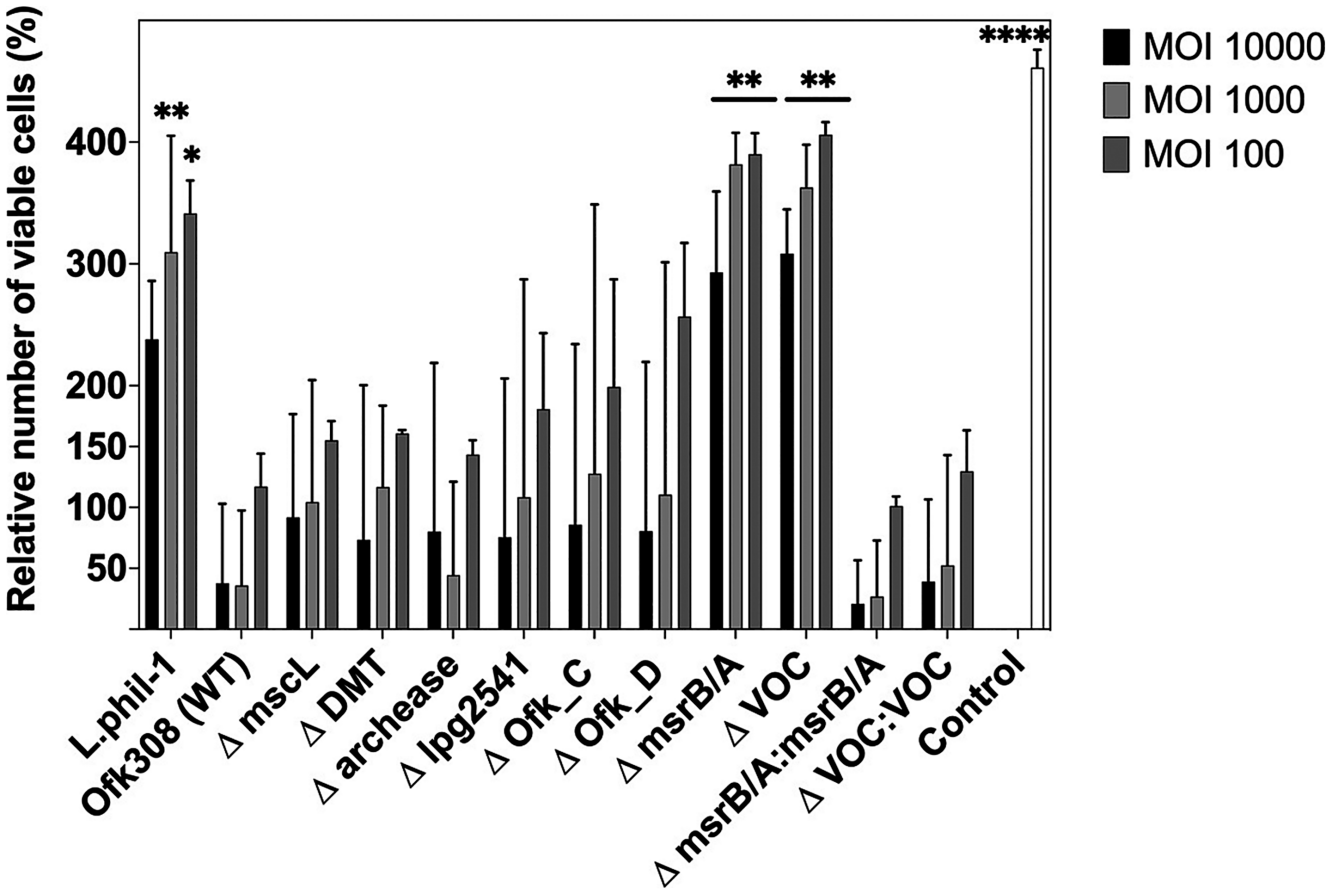
The number of viable CU427 within 48 h after infection at various multiplicities of infection (MOIs). The number of viable CU427 are presented as relative values, with the number of cells at the start set to 100%. Statistically significant differences compared to Ofk308 are indicated by asterisks (*p < 0.05, **p < 0.01, ****p < 0.0001).

To understand gene expression differences between Phil-1 and Ofk308, we performed RNA sequencing analysis. Several genes were identified with relatively high expression levels in Ofk308 compared to Phil-1 (Figure 2 and Table 2). Ofk308 was found to harbor plasmids (Nishida et al., 2017), and some of them have been implicated in no correlation with Ofk308 cytotoxicity (unpublished data). It was strongly suggested that multiple highly expressed genes were located on these plasmids. In this study, we focused on targeting genes located on chromosomes, thereby excluding these genes from further analysis. We then constructed deletion mutant strains of the candidate genes using Ofk308 as the parental strain. Among the 10 candidate genes, Ofk_A and Ofk_B were successfully cloned into pSR47s, but the genes were not disrupted after homologous recombination in BCYE-αcontaining 5% sucrose (data not shown). According to Figure 3, co-culture of *Tetrahymena* and Phil-1 increased the number of surviving CU427 cells. All mutant strains were cytotoxic to CU427 within 48 h, with average relative number of viable cells below 100% at MOI 10000, except for VOC and msrB/A. CU427 viability was dependent on MOI when compared to Ofk308. As previously reported, Ofk308 shows cytotoxicity toward several *Paramecium* strains in an MOI-dependent manner (Watanabe et al., 2016). The relative number of viable CU427 at MOI 10000 showed that Phil-1, ΔVOC, and msrB/A exhibited the highest rates—over 200%—compared to the wild type and other mutant strains. Cytotoxicity observed in drug metabolite transporter (ΔDMT), mscL, archease, lpg2541, Ofk_C, and Ofk_D mutant strains at MOI 10000 was similar to Ofk308 (wild type), with relative number of viable cells falling below 100%. The control, which only included CU427 grown in SPP medium, showed over 400% stably in relative number of viable cells within 48 hpi.

**Table 2:** List of gene candidate used in the study with expression value obtained from RNA-seq.

Gene symbol	Gene description	Notation in this study	Gene expression value (Ofk/Phil-1)
*lpofk_01237*	DMT family transporter	DMT	12.8
*lpofk_02264*	Archease	Archease	15.8
*msrA1*	Methionine sulfoxide reductase B/A reductase	msrB/A	25.7
*fosA1*	VOC family protein	VOC	173.5
*mscL*	mscL	mscL	29.9
*lpofk_02643*	Lpg2541 family Dot/Icm T4SS effector	lpg2541	52.5
*ankX2*	Hypothetical protein	Ofk_A	11.1
*lpofk_01167*	Hypothetical protein	Ofk_B	14.1
*lpofk_02002*	Hypothetical protein	Ofk_C	34.03
*lpofk_00237*	Hypothetical protein	Ofk_D	19.5

### Intracellular growth

**Figure 4:**
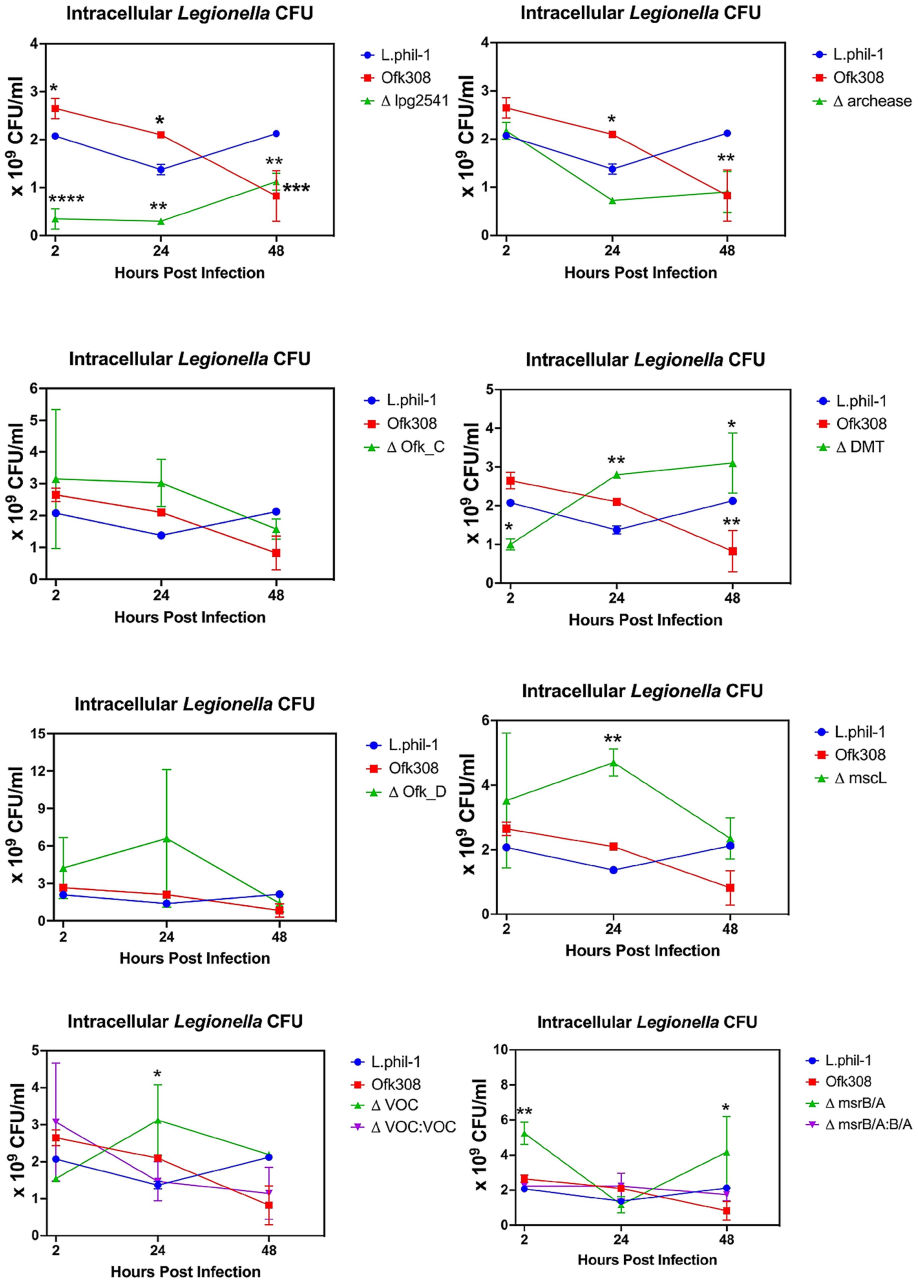
Intracellular growth of *L. pneumophila* within *Tetrahymena thermophila* CU427 cells at a multiplicity of infection (MOI) of 10,000. Statistically significant differences compared to strain Philadelphia-1 (Phil-1) are indicated by asterisks (*p < 0.05, **p < 0.01, ***p < 0.001, ****p < 0.0001).

In this study, we analyzed the ability of *Legionella* to multiply inside the ciliate host CU427. Referring to Figure 4, the CFU number of Phil-1 within CU427 increased over 48 hpi, indicating that Phil-1 can maintain a stable relationship with *Tetrahymena* (Boamah et al., 2017). In contrast, the CFU of Ofk308 decreased within 48 hpi, followed by host cell death. Most of the deletion mutant strains that showed no significant change in cytotoxicity to *Tetrahymena* exhibited a decreasing trend in CFU numbers, similar to Ofk308, while ΔDMT showed a greater increase in CFU numbers than Phil-1. Furthermore, VOC and msrB/A, which had reduced cytotoxicity, exhibited comparable or elevated CFU levels in comparison to Phil-1 at 48 hpi.

### Intracellular bacterial load

**Figure 5:**
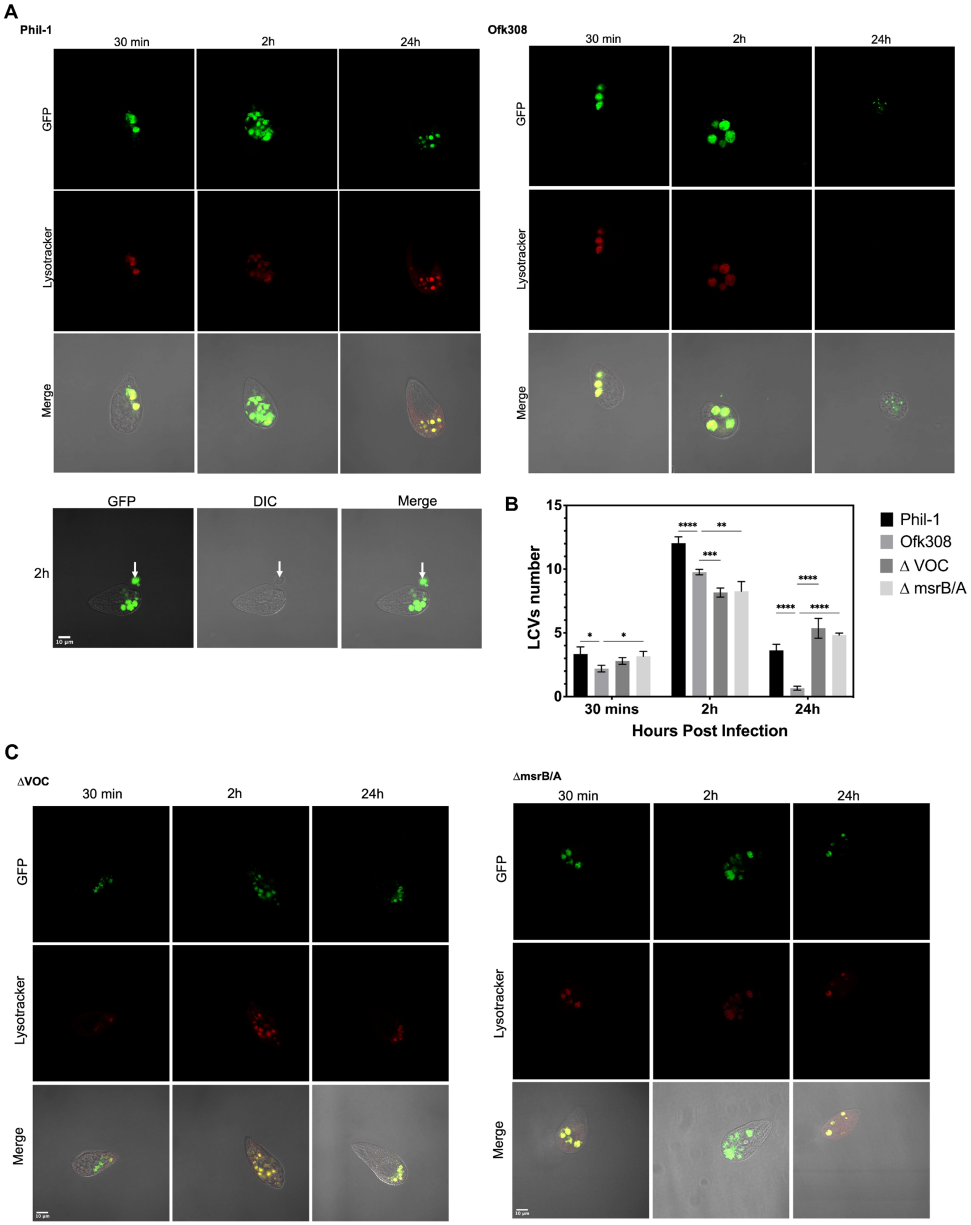
**(A,C)** Localization of *Legionella*-containing vacuoles (LCVs) formed by *L. pneumophila* strains Philadelphia-1 (Phil-1), Ofk308, ΔVOC, and the msrB/A mutant within *Tetrahymena thermophila* CU427 cells at 30 min, 2 h, and 24 h postinfection (hpi) with a multiplicity of infection (MOI) of 10,000. The white arrow indicates the egress of Phil-1 from a vacuole at 2 hpi. **(B)** Quantification of LCVs formed by Phil-1, Ofk308, ΔVOC, and msrB/A per *Tetrahymena* cell. Statistically significant differences compared to Ofk308 are indicated by asterisks (*p < 0.05, **p < 0.01, ***p < 0.001, ****p < 0.0001).

*Legionella-*containing vacuole, within the first 30 min after being engulfed by CU427, appears as a small circular digestive vacuole ranging in size from 3–4 μm at the anterior part of the cell (Figure 5). After 2 h, the average number of LCVs increases from 3 [95% CI: 2.98, 3.61] to 12 [95% CI: 11.35, 12.7] per CU427 cell and becomes localized toward the center and posterior regions. The LCV size remains constant. In contrast, the LCVs of Ofk308 observed 30 min post engulfment were circular but enlarged to 5–7 μm after 2 h, with an average of 2 [95% CI: 1.9, 2.5] to 10 [95% CI: 9.2, 10.2] LCVs per cell (Figure 5). Ofk308 LCVs appear to avoid phagosome maturation within this 2 h period. The cytotoxicity of Ofk308 alters *Tetrahymena* morphology, and no LCVs remain after 24 h. Additionally, *Tetrahymena* infected with Phil-1 begins expelling part of the bacterial pellet within the first 2 h postinfection, while viable bacteria remain inside the pellet. Deletion of VOC and msrB/A results in LCV formation in CU427 in a manner similar to that of Phil-1. Compared to Ofk308, the number of LCVs in VOC and msrB/A mutants is 4 [95% CI: 3.5, 4.5] and 5 [95% CI: 4.5, 5.1] number per CU427 cells exhibits significantly higher at 24 hpi, with no host cell death observed.

## Discussion

RNA-Seq was initially performed to uncover genes with higher expression in the Ofk308 strain (Figure 2 and Table 2), and several candidate genes such as archease, DMT, lpg2541, mscL, msrB/A, and VOC were identified for further construction into deletion mutant strains. Archease is a 16 kDa protein involved in DNA or RNA processing. This was followed by the identification of DMT, a large group of membrane transporters. The next gene, lpg2541, belongs to the Dot/Icm T4SS effector family, contributing to bacterial conjugation, gene transfer, and delivery of virulence factors into host cells. Finally, mscL, along with mscS, forms part of the mechanosensitive channel proteins that provide limited protection against hypoosmotic shock in bacteria (Pivetti et al., 2003; Gomez-Valero et al., 2011; Desai et al., 2014; Tsuchiya et al., 2016). Additional candidates include four hypothetical proteins: Ofk_A, Ofk_B, Ofk_C, and Ofk_D, with unknown functions. We expect that the genes of these candidates contribute during *Legionella* infection within CU427 cells, as further explained.

The biphasic life cycle of *Legionella* has been widely associated with a broad range of protozoan hosts that exhibit highly specific host-pathogen interactions. *Tetrahymena* survives during co-culture with *Legionella* Phil-1, showing an almost twofold increase in number within 2 h postinfection. Naturally, under optimal conditions, *Tetrahymena* demonstrates a rapid growth rate of less than 2 h (Cassidy-Hanley, 2012). This suggests that CU427 may gain mutualistic symbiosis during infection with Phil-1. Interestingly, as previously reported, Ofk308 exhibits cytotoxicity toward several *Paramecium* strains in an MOI-dependent manner (Watanabe et al., 2016), which aligns with our findings that *Tetrahymena* maintains a similar relationship with Ofk308. Furthermore, in our study, CU427 was infected using *Legionella* cultures at the postexponential phase. This growth phase is correlated with increased virulence, cytotoxicity, motility, and the capacity to evade phagosome–lysosome fusion (Molmeret et al., 2004). Thus, our findings provide a more accurate prediction of Ofk308’s cytotoxicity. In contrast to ΔVOC and msrB/A, which significantly altered the relative number of viable CU427 (Figure 3), deletion of archease, DMT, lpg2541, mscL, Ofk_C, and Ofk_D remained cytotoxic to CU427 within 48 hpi. It is possible that these genes are not directly related to Ofk308’s cytotoxicity toward the host, or that Ofk308 may harbor alternative genes that compensate for the deletions, resulting in no change in cytotoxicity in the mutant strains.

Despite *Legionella* growing optimally at 37°C, this bacterium can replicate at temperatures ranging from 25°C–42°C, leading to a broad range of intracellular growth within protist hosts (Richards et al., 2013). A previous study also showed that co-culturing *Legionella* with *Tetrahymena* can increase *Legionella* numbers in environmental water samples (Fields et al., 1984). This aligns with our findings, where the intracellular CFU count of Phil-1 within CU427 increased over 48 h at the optimal growth temperature for *T. thermophila* (30°C). In contrast, due to the death of the *Tetrahymena* host, it is difficult to accurately assess changes in intracellular CFUs for Ofk308 or the deletion mutants that did not reduce cytotoxicity. Notably, each deletion mutant showed a distinct trend in intracellular bacterial numbers. A previous study reported that, in *Legionella* and other Gram-negative bacteria, growth temperature strongly influences gene expression and may trigger pathogenicity (Aroori et al., 2013; Hochstrasser and Hilbi, 2022). However, no studies definitively demonstrate the involvement of our target genes in intracellular growth at 30°C. Future comparative studies across different temperatures may clarify the specific roles of these genes.

Protozoa uptake bacteria through traditional or coiling phagocytosis and confine them to phagosomes, which subsequently fuse with lysosomes (Yang et al., 2022). *Legionella* was taken up into CU427 as a food vacuole and developed into an LCV compartment over time. Intracellular bacterial loads of *Legionella* Phil-1 and Ofk308 were analyzed by counting the LCVs. In Figure 5, a higher number of LCVs containing Ofk308 with enlarged size and the release of Ofk308 into the host cell cytoplasm before host cell lysis indicate that the environmental wild type exhibits greater virulence within CU427. Modified *Legionella,* such as genetic knockdowns or mutants, are known to show reduced growth and egress ability within host cells compared to the wild type (Mraz and Weir, 2022). In Figure 5, the pellet expelled from CU427 after 2 hpi contains viable *Legionella*. This expelled pellet elevates *Legionella’s* virulence, enabling it to persist longer in the environment and form biofilm. Additionally, the expelled pellet is also believed to function as a defensive mechanism of *Tetrahymena* IB against *Legionella* infection or as a food reserve in *T. thermophila* (Hojo et al., 2012; Swart et al., 2018; Kawashiro et al., 2021; Personnic et al., 2021). The fact that the Ofk308 strain is cytotoxic and less able to provide these benefits to *Tetrahymena* hosts suggests significant variation in the relationship between *Legionella* and *Tetrahymena*.

In this study, we continued to focus on the VOC and msrB/A mutant strains. VOCs are members of the 10 metalloenzyme superfamilies, which play roles in various biological reactions, including antibiotic resistance, and are able to utilize metal ions for their catalytic activity. The structural member of VOC is including fosfomycin resistance protein A; *fosA* that is key enzyme for a broad-spectrum antibiotic, fosfomycin. It facilitate the conjugation of glutathione to the exposide ring of fosfomycin that is crucial step for fosfomycin inactivation, thus it conferring resistance (He and Moran, 2011; Ito et al., 2017). *Legionella* is a catalase-positive bacterium; deleting the VOC gene may disrupt its catalytic function (Bandyopadhyay and Steinman, 2000). Despite lack of information about *Legionella* resistance to fosfomycin, the parental strain Ofk308 is originated from water environment posses highly expression in VOC (Table 2, Figure 2). It could be believed that such a horizontal gene transfer from any resistance pathogen is exist and contribute to its survival ability in the environment. Therefore, it may relate to the observation that deleting VOC decreases the intracellular growth of *Legionella* within CU427, exhibiting smaller LCV formation compared to Ofk308 at 2, 24, and 48 hpi. msrB/A is known for its role in protecting cells against oxidative stress. Consisting of two major enzymes, msrA and msrB, it repairs oxidative protein damage by converting methionine sulfoxides (Met-SO) in proteins back to methionine (Met) (Zhao et al., 2010; Cudic et al., 2016). Single deletions of msrB and both msrB/A in other intracellular bacteria, such as *Francisella novicida*, reduce cytotoxicity in mice due to impaired bacterial growth (Saha et al., 2017). Taken together, a similar effect is observed in CU427 after infection by msrB/A, where LCV formation remains small within 24 hpi due to *Legionella* becoming more sensitive to reactive oxygen stress. To confirm these findings, we constructed VOC: VOC and msrB/A: msrB/A, where both genes reduce the relative number of viable CU427 host cells similarly to the parental strain Ofk308 (Figure 3). In addition, the CFU counts of both VOC and msrB/A are statistically reduced within 24 h. Our study demonstrates, for the first time, the roles of VOC and msrB/A in *Legionella* and highlights their significance in the virulence of this pathogen. Understanding the factors involved in the *Legionella* lifecycle within various hosts offers a new perspective on *Legionnaire’s* disease control and prevention.

## Conclusion

The VOC and msrB/A contribute to the intracellular growth of *Legionella* within *T. thermophila*. The significance of these genes would become clearer as their detailed functions and expression regulation mechanisms are elucidated in the future. We highlighted the latest findings in host-pathogen interactions involving *Legionella* and emphasized that maintaining the symbiotic relationship with protist hosts in aquatic ecosystems should be considered to help control the environmental risk of *Legionella* infection.

## Data Availability

The data presented in the study are deposited in the GEO NCBI (https://www.ncbi.nlm.nih.gov/geo/) repository, GEO accession number GSE304426.
